# Perforated caecal diverticulitis mimicking an acute appendicitis: a case report

**DOI:** 10.4076/1757-1626-2-7901

**Published:** 2009-09-09

**Authors:** Lamia Malek, Abdullah Sultan, Mustafa Abbas, Nasser Al-Awadhi

**Affiliations:** 1 Department of Surgery, Farwaniah Hospital, Farwaniah, Al-Jabriah718, 46308Kuwait; 2 Department of Pathology, Farwaniah Hospital, Farwaniah, Al-Jabriah718, 46308Kuwait

## Abstract

Right iliac fossa pain, nausea and vomiting in young adults are common symptoms that require careful surgical assessment with acute appendicitis being a common cause. Uncommonly, other conditions can mimic this presentation such as caecal diverticulitis. This condition is often misdiagnosed due to lack of characteristic features and the commonest method of detection is an intraoperative one. Hereby, we describe a rare case of right iliac fossa pain in a 34-year-old female which mimicked an acute appendicitis. The ability to recognize such condition is vital as its management is different and worse outcome can be prevented by earlier detection and proper management.

## Introduction

Right iliac fossa pain is a common symptom of acute appendicitis, especially in young patients. However, in rare cases it can reflect other pathology which is easy to miss- diagnose. Here, we report a case of caecal diverticulitis in a young female that mimicked appendicitis. The ability to recognize such condition is vital as the management is different.

## Case presentation

A previously fit and healthy 34-year-old middle eastern Kuwaiti female presented with right lower quadrant pain of four days duration radiating to the back. It was associated with nausea and vomiting. She was a house wife and married with four healthy children. She had a recent urinary tract infection treated with antibiotics. She had an intrauterine contraceptive device (IUCD) and gave a history of brownish vaginal discharge. There was no family history of relevance. On examination she was febrile (38 degrees Celsius). Her weight was 90 kilograms and her height was 160 cm. Abdominal examination revealed tenderness and guarding in right iliac fossa (RIF) and right lumber region. Her laboratory investigation showed a leucocytosis with normal urine routine microscopy. Her liver function test and C-reactive protein was normal.

Abdominal ultrasound showed a thin rim of free intra-abdominal fluid in RIF with no masses or localized collection. A trans-vaginal ultrasound showed normal ovaries with an IUCD in situ. We proceeded with diagnostic laparoscopy which revealed serous collection in the right side of the abdomen with a normal-looking appendix. Her caecum was noted to be inflamed. The procedure was then converted to laparotomy through a lower mid-line incision which revealed an inflamed caecal wall with localized perforation. There were no masses palpable in the lumen. We performed an ileocaecal resection with side to side anastomosis.

**Figure 1.**
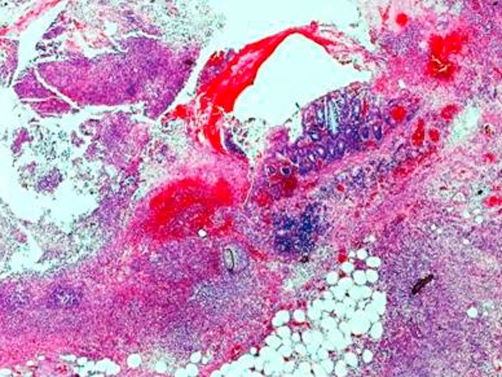
Paraffin-embedded and hematoxylin and eosin-stained microscopic sections showed marked acute inflammatory exudates around the caecum forming the mass. Residual diverticulum was seen within the inflammatory exudates (magnification power × 2.5).

**Figure 2.**
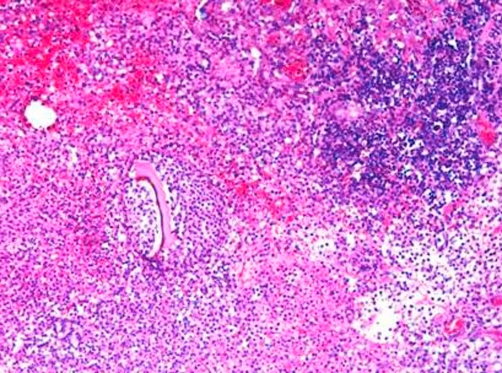
Pieces of colon contents with fecal material were also seen within pericolic fat indicating perforation (magnification power × 10).

Postoperative recovery was smooth with no complications and the patient was discharged home on 6^th^ postoperative day. She remained well three months later. Histologically, there was a marked acute inflammatory exudate around the caecum forming an inflammatory mass (Figure 1). A residual diverticulum was seen within the inflammatory mass with pieces of colon contents and fecal material seen within pericolic fat indicating site of perforation (Figure 2). The colon, terminal ileum and appendix were unremarkable.

## Discussion

Caecal diverticulitis is a rare condition with an incidence ranging between 1:50 to 1:300 of that of appendicitis. It is more common in oriental population [1]. It can be classified into two groups: true (or congenital) and false (or acquired). The true diverticulae are believed to arise from a transient out-pouching of the caecum at 6 weeks gestation and contain all layers of the colonic wall. The false diverticulae are similar in nature to the more common sigmoid diverticulae and contain no muscular layer [2].

The pre-operative diagnosis of right sided colonic diverticulitis is very often difficult. However, the condition can be suspected in patients with longer duration of RIF pain, and in those where the pain starts and become localized in RIF [3]. Radiological investigations that might aid in diagnosis are based on ultrasound and computed tomography (CT) scanning. Ultrasound findings that favours the diagnosis include hyperechoic or hypoechoic outpouching of the right colonic wall and localized circumferential colonic wall thickening at the level of diverticulum [4]. Characteristic CT findings include direct visualization of the diverticulum at the level of maximum circumferential wall thickening [4]. However, the correct diagnosis is often only made intraoperatively at the time of surgical exploration. The surgical treatment of caecal diverticulitis is controversial with the options ranging from conservative management with antibiotics alone to an aggressive resection [5]. A diagnostic laparoscopy was performed in our case as it is a routine procedure done in our hospital for females with right iliac fossa pain. The appendix was normal and the caecum was inflamed. If an inflamed caecal diverticulum is identified at laparoscopy, this can be managed laparoscopically if the operating surgeon has the required expertise. If not, the surgeon can simply treat with antibiotics alone or converted to an open procedure, as we have done. In our case we have converted to an open procedure to further evaluate the caecum [1]. Intraoperative diagnosis of caecal diverticulitis is not always clear. Correct diagnosis can only be made in 60%-70% of cases. Some authors recommend careful mobilization of the caecum and careful palpation to detect the ostium of the diverticulum [5]. Controversies exist regarding appropriate operative management for right colonic diverticulitis encountered in surgery for presumed appendicitis. Some surgeons advocate surgical resection (as we have done in our case) while others suggest a conservative management. However, In the presence of complications, such as free perforation or localized abscess formation, colectomy should be considered [6].

In conclusion, we hereby present a rare case of caecal diverticulitis that mimicked acute appendicitis. The ability to recognize such condition is vital to dictate the appropriate therapy and prevent a worse clinical outcome.

## Abbreviations

CT: computed tomography
IUCD: intrauterine contraceptive device
RIF: right iliac fossa
